# The Predictive Value of Umbilical Cord Interleukin-6: Implications for Neonatal Care—A Narrative Review of Current Evidence and Future Perspectives

**DOI:** 10.3390/life15111727

**Published:** 2025-11-09

**Authors:** Diana Iulia Vasilescu, Adriana Mihaela Dan, Andreea Raluca Gogoncea, Sorin Liviu Vasilescu, Monica Mihaela Cîrstoiu

**Affiliations:** 1 Doctoral School, “Carol Davila” University of Medicine and Pharmacy, 020956 Bucharest, Romania; diana.vasilescu@umfcd.ro; 2 Department of Neonatology, Emergency University Hospital Bucharest, 050098 Bucharest, Romania; 3 Faculty of Medicine, “Carol Davila” University of Medicine and Pharmacy, 020956 Bucharest, Romania; drsorinvasilescu@gmail.com (S.L.V.); monica.cirstoiu@umfcd.ro (M.M.C.); 4 Department of Neonatology, “Grigore Alexandrescu” Clinical Emergency Children Hospital Bucharest, 011743 Bucharest, Romania; 5 Department of Obstetrics and Gynaecology, Emergency University Hospital Bucharest, 050098 Bucharest, Romania

**Keywords:** interleukin-6, umbilical cord blood, biomarker, fetal inflammatory response syndrome, early onset neonatal sepsis, neonatal outcomes, morbidity

## Abstract

(1) Background: The assessment of neonatal health and prognosis is one of the most critical areas in pediatric medicine. Intrauterine inflammation and the fetal inflammatory response syndrome (FIRS) are increasingly recognized as major determinants of neonatal morbidity. Interleukin-6 (IL-6), measured in the umbilical cord (UC) blood, has emerged as a promising biomarker, reflecting both intrauterine conditions and early neonatal risk. This narrative review aims to synthesize current evidence on the predictive value of umbilical cord blood IL-6 for neonatal outcome, including sepsis, respiratory distress, hypoxic–ischemic encephalopathy (HIE) and mortality. (2) Methods: A comprehensive literature search was conducted in PubMed, Scopus, and Web of Science. Studies reporting umbilical cord IL-6 levels in relation to neonatal outcomes were analyzed and summarized narratively. (3) Results: Evidence consistently indicates that elevated umbilical cord IL-6 is associated with early-onset neonatal sepsis (EONS) and respiratory complications, and provides prognostic insight into neurological outcomes, even though results are influenced by gestational age (GA), mode of delivery, and the presence of chorioamnionitis. (4) Conclusions: UC IL-6 represents a valuable early biomarker for neonatal risk stratification and supports clinical decision-making. Future research should prioritize assay standardization, reference interval development, and prospective multicenter studies to validate its integration into routine neonatal care.

## 1. Introduction

Neonatal mortality and morbidity remain pressing global health challenges that have a significant impact on public health systems and the well-being of families, in different socio-economic settings [1]. The assessment of neonatal health and prognosis is one of the most critical areas in pediatric medicine. A useful tool for prognosis and early detection of diseases proved to be the inflammatory biomarkers [2]. The integration of biomarker research into neonatal care not only promises to improve individual patient outcomes but also has the potential to enhance public health strategies aimed at reducing neonatal morbidity and mortality rates. Biomarkers can guide taking clinical decision, help tailor interventions and inform parents about future health trajectories of their infants [3].

One of the most studied biomarkers in recent decades is the cytokine IL-6. IL-6 is a multifunctional pro-inflammatory cytokine that plays a crucial role in immune response [4]. When tissue damage or inflammation due to infections or injuries occurs, IL-6 synthesis is promptly induced, peaking 2–6 h afterwards [5,6]. IL-6 serves as a vital biomarker in neonatology, particularly concerning the prognosis of newborns. In the delicate balance of the neonatal immune system, IL-6 can influence clinical outcomes in various conditions, including infections, inflammatory diseases and other complications [7]. Assessing IL-6 levels in UC blood offers a unique window into the inflammatory environment the newborn was exposed to, helping clinicians anticipate potential risks right from the moment of birth [8]. Elevated IL-6 levels in cord blood capture the inflammatory environment, establishing a connection with the concept of FIRS, a condition associated with early morbidity and long-term neonatal complications [9].

In this review, we aim to summarize existing knowledge on the role of UC IL-6 as a predictor of neonatal outcomes, including sepsis, respiratory morbidity and neurodevelopmental prognosis, while also highlighting the challenges and future perspectives for its clinical application.

## 2. Materials and Methods

**Figure 1:**
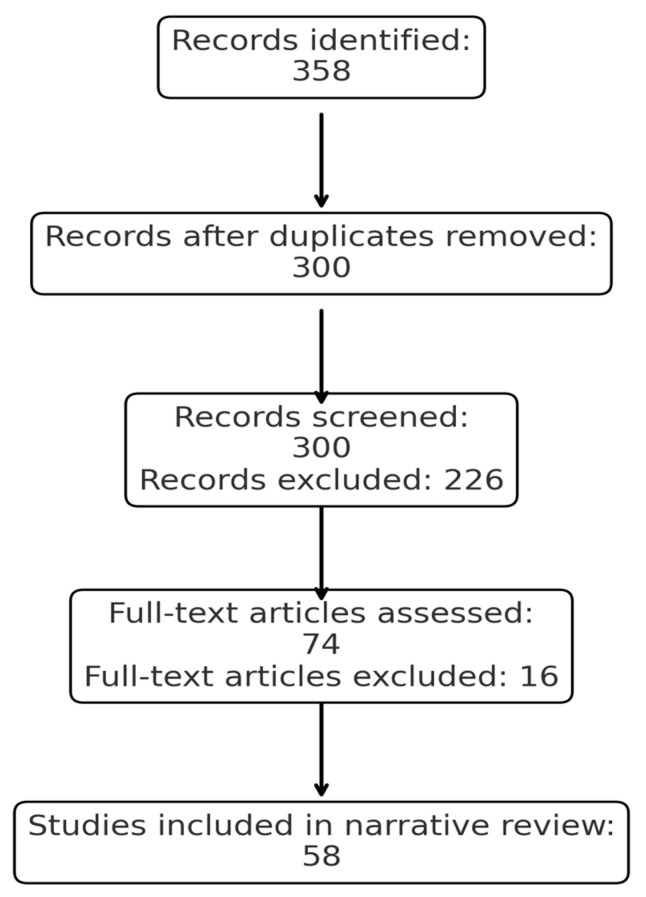
PRISMA flow diagram of the study selection process.

We conducted a comprehensive literature search in PubMed/MEDLINE, Scopus and Web of Science, last updated on 15 September 2025, focusing on studies published between 2000 and 2025. The following search terms and their combinations were used: *“umbilical cord blood”*, *“interleukin-6”*, *“IL-6”*, *“fetal inflammatory response syndrome”*, *“neonatal outcomes”*, *“sepsis”*, *“respiratory distress”*, and *“neurodevelopment”*. All retrieved records were imported into Mendeley Reference Manager for automatic and manual deduplication. We screened titles and abstracts for relevance, followed by full-text assessment. A total of 358 records were identified, of which 58 duplicates were removed. 300 records underwent title/abstract screening, and 74 full-text articles were reviewed. Finally, 58 studies met the eligibility criteria and were included in the narrative synthesis. We included original studies (clinical trials, cohort, case–control, cross-sectional) reporting IL-6 levels in umbilical cord blood and their association with neonatal outcomes, review articles and meta-analyses that provided additional context, as detailed in Figure 1. We excluded research published in languages other than English, animal studies, case reports, conference abstracts, and articles without full-text access. Relevant data were extracted regarding study design, population, IL-6 measurement methods, cutoff values, and reported neonatal outcomes. Given the narrative nature of this review, findings were synthesized qualitatively rather than pooled quantitatively.

## 3. Definition and Role of IL-6 in Immune Response

### 3.1. Biological Role of IL-6

IL-6 is a pleiotropic cytokine with both pro-inflammatory and anti-inflammatory properties. It plays a central role in immune homeostasis, hematopoiesis and the acute-phase response [10]. Monocytes, macrophages, T cells, B cells, endothelial cells and fibroblasts are the various cells that produce IL-6, in response to infection, injury and other inflammatory stimuli [10]. IL-6 exerts its effects through two distinct signaling mechanisms. Firstly, the classic signaling, involves binding to a receptor for IL-6 located on the cell membrane—the IL-6 receptor (IL-6R) and gp130 [5]. Secondly, the trans-signaling, in which IL-6 binds to a soluble form of IL-6R (sIL-6R), allowing activation of cells that do not express IL-6R via the ubiquitously expressed gp130 receptor [5]. Classic signaling is generally associated with regenerative and anti-inflammatory functions, while trans-signaling is the one involved in chronic inflammation and sepsis [5,11].

IL-6 is located in the fetal–maternal tissues and one of its roles is to regulate placental and fetal growth [12]. At the end of pregnancy, it coordinates the biochemical, immunological and physiological changes necessary for the survival of the mother and fetus [13]. IL-6 is also implicated in the pathogenesis of intrauterine inflammation and FIRS, the latter being regarded as the fetal counterpart of the systemic inflammatory response syndrome seen in adults [14]. FIRS is characterized by elevated fetal cytokines and was first described in 1998, by Gomez et al. [15]. It was then defined as an umbilical vein concentration of IL-6 above 11 pg/mL, and this value is still the threshold in today’s definition, together with the histologic proof of inflammation in the placenta [15,16,17]. FIRS is mainly caused by intrauterine infection or chronic hypoxia in the absence of infection [18,19]. EONS, bronchopulmonary dysplasia (BPD), intraventricular haemorrhage (IVH), periventricular leukomalacia (PVL), respiratory distress syndrome (RDS) and neonatal death are associated with FIRS [17,20,21].

Newborns, and preterm infants in particular, display a distinct immunological phenotype characterized by a high IL-6 to TNF-α production ratio both in vitro and in vivo models [22]. This pattern reflects a shift of the neonatal immune response toward Th2-type immunity and limited Th1 cytokine production, which may reduce harmful inflammation but also compromises antimicrobial defense [22]. This imbalance may partly explain the high susceptibility of newborns to bacterial infections, such as group B streptococcus sepsis [23].

### 3.2. UC Blood IL-6

Sampling the UC at birth is a non-invasive, easy and practical alternative for assessing the status of the newborn. Several studies have demonstrated a correlation between IL-6 concentrations in amniotic fluid and those in UC blood, particularly in cases of prolonged preterm rupture of membranes (PPROM) [24,25]. In pregnancies complicated by intraamniotic inflammation (IAI) and/or microbial invasion of the amniotic cavity (MIAC), IL-6 levels in cord blood were significantly higher. Musilova et al. found that women with both IAI and MIAC had median UC IL-6 concentrations of 32.6–39.4 pg/mL, compared to 5.8 pg/mL in those without inflammation or infection, and this was associated with very high rates of FIRS (67–78%) [24].

In addition, the level of IL-6 concentration from the UC proved to be proportional with the level of inflammation found histologically. For example, the stage of the UC inflammation demonstrated a statistically significant direct connection with the concentration of IL-6 from UC blood [26]. Consequently, assessing the inflammatory markers from the cord blood of the infant may be as useful as analysing the UC samples under the microscope, but more rapid and with reduced costs [26].

In terms of thresholds of IL-6 from UC blood, the exact cut-off point may vary, depending on the population and measurement method. As mentioned above, the UC blood IL-6 concentration exceeding 11 pg/mL, is a value commonly cited as diagnostic for FIRS, thus associated with poor neonatal outcomes [1]. However, the values are highly dynamic and context dependent.

**Figure 2:**
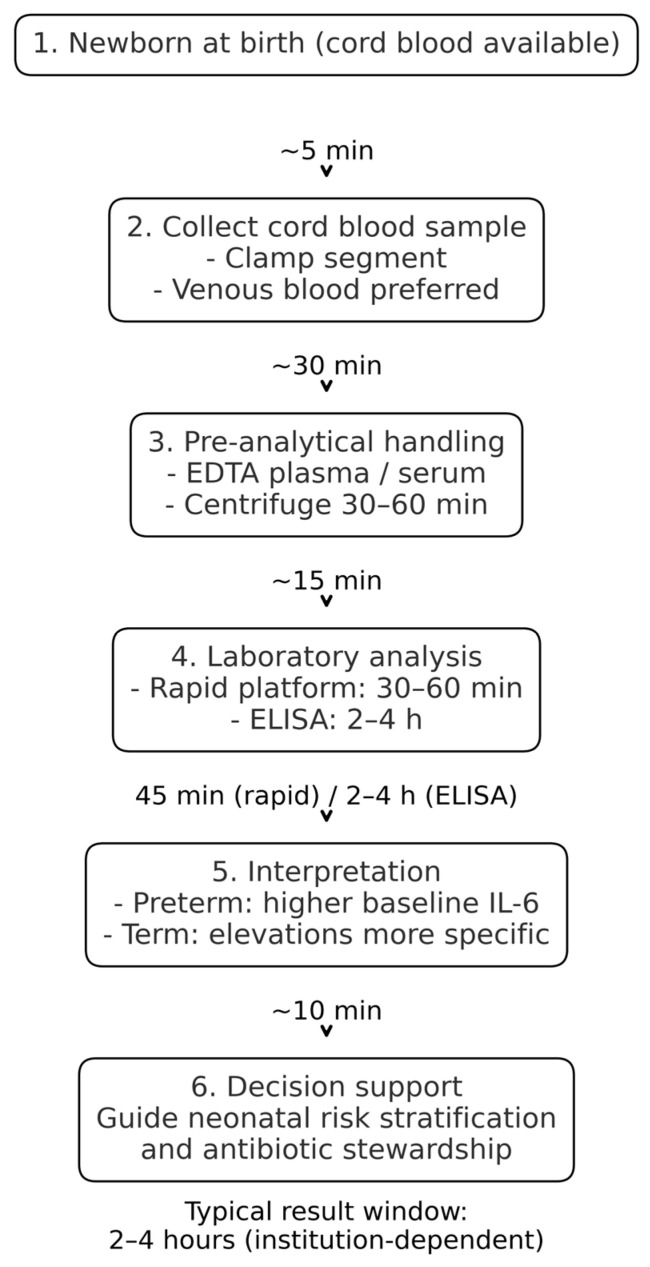
Workflow for UC IL-6 measurement, with estimated timelines for sample collection, processing, analysis, and result reporting.

The collection of UC blood for IL-6 determination at birth offers the unique advantage of providing an actionable result within approximately two hours, thereby enabling early identification of newborns at risk. The workflow outlined in Figure 2 illustrates the estimated timelines of UC IL-6 measurement to provide clinically relevant information within two hours after birth, enhancing neonatal risk assessment and guiding early interventions.

It is important to note that achieving this turnaround time depends on the availability of on-site immunoassay platforms, trained laboratory staff and predefined sample handling pathways. In settings where samples require transport to off-site laboratories or where IL-6 is not validated as on-call test, the turnaround time may be longer. Therefore, implementation requires workflow alignment at the institutional level.

**Figure 3:**
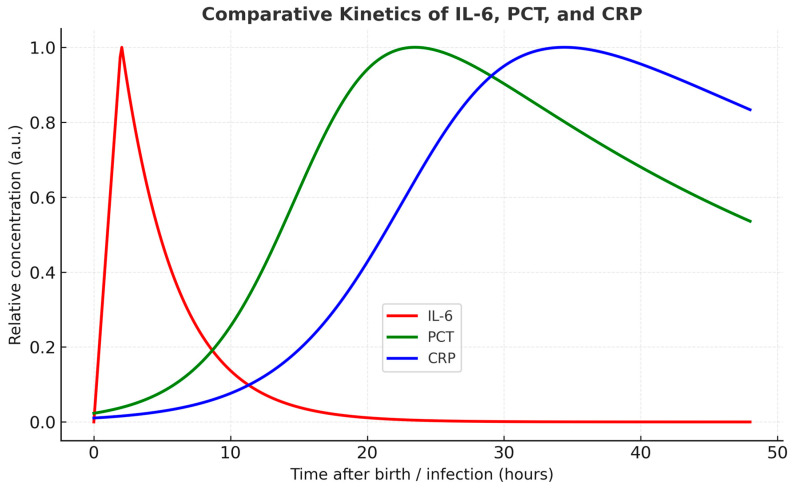
Temporal profiles of IL-6, PCT, and CRP. IL-6 rises rapidly with a peak at ~2 h and declines within 24 h. PCT increases progressively from ~6–12 h, peaking at 18–24 h, followed by a gradual decline. CRP shows a slower and later increase, peaking between 24 and 48 h. Legend is displayed within a boxed area below the curves.

Understanding the distinct temporal profiles of commonly used inflammatory markers is essential for accurate diagnosis and clinical decision-making in neonatal care. The diagnostic value of IL-6 becomes clearer when its kinetics is compared with those of C reactive protein (CRP) and procalcitonin (PCT) [27,28]. A comparative view of IL-6, CRP and PCT dynamics in peripheral blood after birth highlights the unique advantage of IL-6 as the earliest available biomarker [27,29]. The dynamic profiles of IL-6, CRP, and PCT are depicted in Figure 3, emphasizing their different diagnostic windows.

## 4. IL-6 and Neonatal Outcome—Evidence Overview

### 4.1. EONS

The most studied relationship involving IL-6 and FIRS is with EONS. Neonatal sepsis is a global health problem contributing significantly to neonatal morbidity and mortality. EONS occurs in newborns within the first 72 h of life [30]. The diagnosis of EONS is one of the most difficult challenge for a neonatologist [31]. The gold standard for diagnosis of EONS, blood culture, takes 24–72 h for a reliable result, which can be, furthermore, influenced by the prenatal use of antibiotics [29]. The level of IL-6 is considered to be the earliest biomarker for the presence of an infectious process in newborns [32].

Multiple studies show that UC IL-6 is a valuable biomarker for identifying newborns at risk for EONS [33,34,35]. In a prospective cohort study, Cernada et al. demonstrated that cord blood IL-6 had a significantly higher diagnostic performance than CRP. Using a cut-off of 255.87 pg/mL, IL-6 achieved a sensitivity of 90%, specificity of 87.4%, and an area under the ROC curve (AUC) of 0.88, outperforming CRP (AUC—0.70). The likelihood ratios further supported its predictive value, suggesting a strong ability to both rule in and out infection in at-risk newborns [33].

Similarly, Basu et al. found IL-6 in the UC blood to be a highly sensitive and specific marker for culture-proven EONS in newborns with antenatal risk factors. With a lower cut-off of 40.5 pg/mL, IL-6 yielded 92.3% sensitivity, 90.5% specificity, and an AUC of 0.959, far surpassing conventional sepsis screening markers such as CRP, total leukocyte count and absolute neutrophil count. This study highlighted IL-6’s ability to rise earlier than CRP and its potential utility in minimizing unnecessary antibiotic exposure in uninfected newborns [34].

Another important study conducted by Hatzidaki et al. found that IL-6 concentrations in maternal, UC and neonatal blood were significantly higher in newborns with EONS compared with those without EONS (*p* < 0.001) with a cut-off concentration of IL-6 in UC blood of 108.5 pg/mL for EONS (sensitivity 95%, specificity 100%, positive predictive value 100%, and negative predictive value 97.4%) [36].

A more recent cohort study undertaken by Yuan et al. reinforced these findings in premature infants. ROC curve analysis demonstrated that IL-6 levels in the UC blood above 250.5 pg/mL predicted EONS with 90% sensitivity and 82% specificity (*p* < 0.001), an AUC of 0.876, with a confidence interval of 0.753–0.999. These results validate IL-6’s diagnostic strength across different populations and also underline its high accuracy as a diagnostic marker among preterm infants, where early signs of infection are often ambiguous [37].

Fadilah et al. demonstrated that newborns with elevated UC IL-6 levels were 5.54 times more likely to develop EONS than those with normal IL-6 levels [RR 5.54 (95%CI 1.68 to 18.25); *p* = 0.016] [38]. Another research conducted by Yoon et al. found that an IL-6 concentration of ≥17.5 pg/mL in UC plasma had a sensitivity of 70% and specificity of 78% for detecting funisitis, which was significantly associated with EONS [39]. Another study from 2014, on 218 premature infants, 30 of which were diagnosed with EONS, reported even higher accuracy using a cut-off of 15.85 pg/mL, achieving 73.7% sensitivity and 84.2% specificity, and noted further improvement when IL-6 was combined with PCT [40].

The 2024 systematic review and meta-analysis conducted by van Leeuwen et al. synthesized data from multiple trials and estimated pooled sensitivity and specificity for IL-6 at 83% and 87%, respectively. Although variability in study design and sepsis definitions existed, the overall evidence supports IL-6 as one of the most promising standalone biomarkers currently available [41].

Another systematic review evaluated 31 studies with over 3200 newborns and found that IL-6 cut-offs above 30 pg/mL were commonly used. Median diagnostic performance across studies was 83% sensitivity and 83.3% specificity. Notably, the diagnostic value was higher in preterm infants and when cord blood was used rather than peripheral samples. While IL-6 was highly sensitive on its own, combining it with CRP increased overall sensitivity but reduced specificity, reflecting a trade-off between early detection and false positive [30].

**Figure 4:**
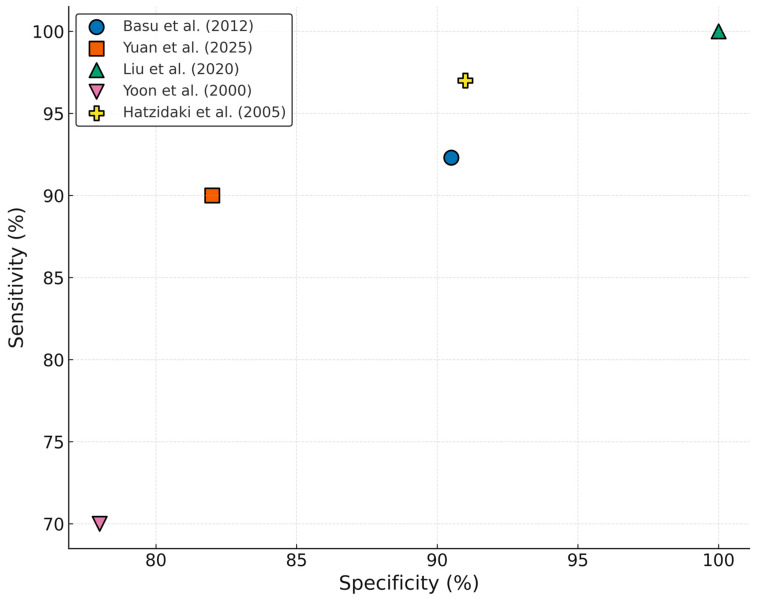
Diagnostic performance of UC IL-6 in neonatal sepsis studies [29,34,36,37,39].

Taken together, these studies consistently highlight the diagnostic value of UC IL-6 in EONS, supporting its role as a reliable biomarker. The key findings are illustrated in Figure 4.

Table 1 summarizes selected studies that have evaluated proposed cut-off values of UC IL-6 for the diagnosis of EONS. These findings highlight the variability of thresholds across populations, GA and a comparison of the diagnostic value of IL-6 with other commonly used markers.

The diagnostic performance of IL-6 varies depending on the sample source and timing of collection. Most studies assessed IL-6 in UC blood obtained at birth, where IL-6 levels best reflect the fetal inflammatory response [30,31,34,35]. One study also measured IL-6 in postnatal peripheral blood, where levels may decline rapidly and are more time-dependent [37]. These differences are summarized in Table 2, which highlights that UC blood sampling generally provides the most reliable early diagnostic window for EONS.

Overall, while UC IL-6 demonstrates significant potential as an early biomarker for neonatal sepsis, the heterogeneity of reported cut-off values underscores the need for further large-scale, standardized studies to establish clinically reliable thresholds.

### 4.2. Respiratory Complications

Another correlation identified for IL-6 concerns neonatal respiratory complications. Most studies focus on the possibility of using the level of UC IL-6 to predict the development of RDS and BPD in premature infants.

A prospective cohort study from 2014 on 150 preterm infants under 30 weeks of gestation found that elevated IL-6 in cord blood was associated with moderate or severe BPD, especially in small for GA newborns [43]. In a more targeted investigation, Ozalkaya et al., analysed a population of 84 preterm newborns, who were divided into a FIRS group (defined by a value of IL-6 from umbilical vein blood above 11 pg/mL) and a non-FIRS group, who served as control. The study reported that IL-6 above 26.7 pg/mL predicted RDS with a sensitivity of 70% and a specificity of 80%. ROC analysis supported these findings with AUC values of 0.81 for RDS [44].

One larger cohort study, on 224 newborns, involving various cytokines profile from the UC blood, proved a statistically significant correlation between the concentration of UC IL-6 with RDS (*p* < 0.0007) and chronic lung disease (CLD) (*p* < 0.0001) [3]. However, Satar et al. evaluated 83 premature infants with and without PPROM and they showed that while maternal serum IL-6 was significantly higher in the PPROM group (*p* < 0.01), there was no difference between UC IL-6 levels between groups (*p* > 0.05), and no association was observed between IL-6 and RDS or BPD [45]. Sorokin et al. assessed 400 preterm newborns in a secondary analysis of a randomized corticosteroid trial, reporting that IL-6 levels above the 75th percentile were linked to RDS and CLD, although these associations lost significance after adjusting for GA (adjusted *p* > 0.05) [46].

Overall, the evidence indicates a significant association between elevated UC IL-6 levels and the occurrence of respiratory complications in newborns. The main findings are presented in Table 3.

### 4.3. Neurological Outcomes

Elevated UC IL-6 was also studied as a marker of adverse neurological outcome in newborns, particularly those born prematurely. A level of IL-6 from UC above 107.7 pg/mL was demonstrated to increase 30 times the risk of PVL [47]. These findings support the hypothesis that FIRS, marked by elevated fetal IL-6, contributes to cerebral white matter injury, initiated in utero.

A study by Musilova et al. conducted in 2018 showed that the presence of FIRS—determined by IL-6 levels in cord blood along with placental histopathology—was associated with an increased risk of IVH grades I and II, further emphasizing IL-6 as a marker of systemic vulnerability that could predispose brain injury in the presence of other risk factors [24]. Only a small number of studies have explored the link between cytokines and central nervous system injury in term infants. Chiesa et al. found that cord blood IL-6 levels increased considerably after birth asphyxia, and that these increases were more pronounced in the newborns with severe clinical course and poor prognosis, even in the absence of infection [6].

A more recent, case–control study analysed immuno-inflammatory biomarkers from the UC in a total of 150 term newborns, divided into 3 groups: 50 newborns had birth asphyxia and HIE, 50 newborns had birth asphyxia without HIE, and 50 newborns were included in the control group. Regarding IL-6, it was discovered that IL-6 levels were higher in all HIE cases versus controls (*p* = 0.008). There was an association with the degree of HIE and levels of IL-6 (*p* = 0.002). IL-6 levels were higher in HIE stage II/III according to Sarnat criteria versus controls (*p* = 0.0002). IL-6 in the UC predicted HIE stage II/III with an AUC of 0.827. IL-6 levels were higher (*p* = 0.007) in HIE stage II/III versus HIE stage I [48].

Nevertheless, not all existent studies report a correlation with neurological outcomes. A large multicentre cohort study on 400 newborns found that IL-6 above the 75th percentile was not significantly associated with neurodevelopment impairment at 36 to 42 corrected age (neurodevelopment impairment was defined using Bayley Scales of Infant Development: Bayley Mental Development Index (MDI) and Psychomotor Developmental Index (PDI) scores <70) [46].

Wolfsberger et al. investigated the effects of FIRS (defined using the classic definition of UC IL-6 >11 pg/mL) on cerebral oxygenation and short-term brain injury in preterm newborns. Using near-infrared spectroscopy, the authors found that newborns with FIRS exhibited significantly altered cerebral oxygen extraction within the first 15 min after birth, indicating early hemodynamic changes in the brain. However, despite these alterations, there was no significant difference in the incidence of cerebral injury (e.g., IVH or PVL) between FIRS and non-FIRS groups during early neonatal imaging. These findings suggest that while FIRS and elevated IL-6 may affect cerebral physiology immediately after birth, they do not necessarily translate into detectable structural brain injury in the short term [49].

Overall, current evidence indicates that elevated UC IL-6 levels may serve as a prognostic indicator of adverse neurological outcomes in both term and preterm newborns. The principal findings are summarized in Table 4. However, data remain limited, and further research is warranted.

### 4.4. Necrotizing Enterocolitis (NEC)

There are no studies specifically dedicated to the relationship between elevated UC IL-6 and NEC, but there are several studies that include NEC among the neonatal adverse outcomes and the predictive value of IL-6. Goepfert et al. found that IL-6 above 11 pg/mL in cord blood was significantly associated with NEC in preterm newborns, although specific odds ratios for NEC alone were not isolated [47].

In contrast, a prospective observational study of newborns born to mothers with PPROM found that among infants who developed NEC, IL-6 levels from UC were not significantly different from those who did not. Moreover, IL-8 proved to be a more relevant inflammatory marker for NEC than IL-6 (*p* < 0.05) [45]. Another research on the association of cord blood levels of IL-6 and NT-proBNP with perinatal variables of premature infants below 32 weeks of gestation proved that none of the infants who later developed NEC had elevated IL-6 in the UC blood (*p* < 0.01) [50]. In 2014, Sorokin et al. found that IL-6 levels above 75th percentile were not associated with NEC in the full cohort of 400 newborns. However, when studied in a subgroup of premature infants below 32 weeks of gestation, a significant association emerged (*p* < 0.05) [46].

Current evidence investigating the association between IL-6 and NEC is limited and does not demonstrate a strong link, highlighting the need for further research. The key findings are summarized in Table 5.

### 4.5. Mortality

Elevated UC IL-6 and FIRS have been strongly correlated with increased neonatal mortality. Multiple studies have explored this relationship, demonstrating that IL-6 is not just a marker of inflammation, but also a potential predictor of survival outcomes in newborns.

In a pivotal study in 2015, 160 preterm newborns were divided into a FIRS group (cord blood IL-6 > 11 pg/mL) and a non-FIRS group. The mortality rate in the FIRS group was 19.3%, significantly higher than 1.9% mortality observed in the non-FIRS group, representing a tenfold increased risk (*p* < 0.001). UC blood IL-6 concentration > 37.7 pg/mL was found to be predictive of death, with 78.6% sensitivity and 60% specificity [44]. Similarly, Hofer et al. found that FIRS was significantly associated with neonatal death in a cohort of 176 preterm infants. Among the 62 infants classified as FIRS (IL-6 > 11 pg/mL), the correlation coefficient between UC IL-6 levels and adverse outcomes, including mortality, was r = 0.411 (*p* < 0.001), and even stronger for those born before 32 weeks of gestation (r = 0.481, *p* < 0.001) [51].

Further support for the mortality risk comes from Nomiyama et al., who analysed 330 preterm infants and stratified them by presence of FIRS and maternal inflammation. They reported that FIRS, both with and without accompanying maternal inflammation, was significantly associated with composite adverse neonatal outcomes including death. The adjusted odds ratios for mortality and morbidity ranged from 6.84 to 7.17 depending on inflammatory status, reinforcing the dangerous synergy between fetal and maternal inflammation [17].

Not all studies found a correlation between increased risk of mortality and IL-6. For example, Satar et al., in a smaller cohort of 83 newborns (42 with PPROM), reported no significant difference in cord IL-6 levels between survivors and non-survivors, nor a strong correlation between IL-6 and neonatal death [45]. These findings demonstrate a potential association between elevated IL-6 levels and increased neonatal mortality; nevertheless, evidence remains scarce and inconclusive, warranting further investigation. The main findings are presented in Table 6.

## 5. Challenges and Controversies

While numerous studies support the diagnostic and prognostic value of UC IL-6, important challenges and controversies persist, including methodological heterogeneity, variable cut-off values, and limited standardization across clinical settings.

Our research highlights the potential clinical utility of UC blood IL-6 measurement immediately at birth, providing a valuable time-window for early risk stratification and guiding antibiotic stewardship in newborns at risk of EONS. When considered alongside conventional markers such as CRP and PCT, IL-6 appears to add additional diagnostic value, supporting the rationale for a combined biomarker approach in early neonatal sepsis evaluation. Nevertheless, one major clinical limitation for the use of UC IL-6 as a predictor for sepsis is the variable cut-off values reported in the studies. Reported thresholds in the literature range from 15 to 250 pg/mL, also depending on GA [30,37,40]. This makes it difficult to standardize UC IL-6 as a diagnostic tool.

Likewise, regarding respiratory outcomes, several studies highlight the utility of IL-6 as a potential early marker of inflammatory-driven respiratory morbidity in newborns, though variations in study design, population characteristics, and cut-off thresholds underscore the need for standardized protocols and further validation [43,44,45,46]. The discrepancy across studies that focused on the level of UC IL-6 and neurodevelopment outcomes suggests that while IL-6 may signal acute inflammatory states linked to brain vulnerability, it may not independently predict long-term outcomes without additional insults or co-factors. IL-6 as a biomarker may serve as more of an early warning signal of vulnerability rather than a direct predictor of neurological damage. Regarding NEC, GA may modulate the relationship between inflammatory markers and NEC risk, with IL-6 becoming more predictive in extremely preterm infants [46,47].

Several studies show a powerful association between increased risk of death, FIRS and elevated IL-6 from the UC [17,51]. However, there is a study from 2008 that showed no significant difference in cord IL-6 levels between survivors and non-survivors [45]. However, this study may have been underpowered to detect mortality effects due to sample size limitations and the relatively low baseline mortality rate.

An interesting aspect is the difference in UC IL-6 levels between newborns born vaginally and those delivered by cesarean section, a variation that may reflect the inflammatory response triggered by the stress of labor and the different perinatal exposures associated with each mode of delivery [52]. Multiple studies have explored the impact of the mode of delivery on UC IL-6 concentrations. In one cohort, cord IL-6 levels were notably higher in vaginally delivered newborns compared to those born by elective cesarean section (5.15 ± 7.6 vs. 2.93 ± 0.49 pg/mL; *p* = 0.09), suggesting a trend toward increased in utero inflammatory activation during vaginal delivery [52]. Other research has demonstrated a statistically significant elevation in cord IL-6 levels following spontaneous vaginal delivery versus elective cesarean (*p* < 0.0001) [53]. Furthermore, cord IL-6 concentrations were shown to increase approximately four-fold during labor compared to non-laboring cesarean deliveries, with an additional six-fold rise in cases with histologic chorioamnionitis [54,55].

To further complicate the interpretation of UC IL-6 levels, recent meta-analyses and systematic reviews have consistently identified significant heterogeneity among studies with respect to cut-off values and GA of the newborns. Several studies have demonstrated that UC IL-6 concentrations vary according to GA, reflecting both maturational differences in the immune response and distinct perinatal exposures. In preterm infants, particularly those born before 32 weeks of gestation, IL-6 levels tend to be higher even in the absence of confirmed infection, largely due to intrauterine inflammation and conditions such as PPROM [56]. This elevation has been interpreted as a consequence of the heightened susceptibility of preterm newborns to inflammatory stimuli, which may result in elevated baseline cytokine concentrations.

In contrast, term newborns generally exhibit lower baseline UC IL-6 concentrations, and elevations are more often associated with acute infectious or inflammatory insults occurring close to delivery. Comparative immune profiling has shown that pro-inflammatory cytokine responses, including IL-6, are less pronounced in preterm compared to term infants under non-infectious conditions [57].

Taken together, these findings suggest that diagnostic cut-off values for UC IL-6 should be interpreted in the context of GA to avoid both false-positive and false-negative results. Additionally, cut-offs obtained from ELISA-based platforms with lower detection limits tend to be lower and more precise than those reported using older immunoassay techniques. For these reasons, cut-offs should be interpreted in the context of the population characteristics and the assay type.

To enhance interpretability while acknowledging the variability of reported thresholds, we summarized representative studies according to gestational age and assay platform (Table 7). Because most studies derived cut-off values within their own cohorts and only one validated its threshold externally, these values should be viewed as descriptive rather than clinically prescriptive. This supports our conclusion that standardized, gestational age-specific reference intervals remain needed before UC IL-6 cut-offs can be operationalized in routine practice.

## 6. Future Directions

Recent advancements in understanding IL-6’s role in neonatal health have also highlighted its potential as a prognostic marker for conditions beyond immediate inflammatory responses. For instance, studies have indicated that elevated UC IL-6 levels can serve as predictors for long-term developmental outcomes in preterm infants, including cognitive and motor skills, suggesting that early intervention strategies could be tailored based on IL-6 assessments to improve not just immediate health but also future quality of life [30]. Future studies should further elucidate the mechanistic interplay between IL-6 and IL-1 in the context of neonatal inflammation and cerebral injury. Evidence suggests that IL-1 acts as a main cytokine responsible for cerebral injury through the induction of vasospasm and microvascular dysfunction, whereas IL-6 may represent an upstream mediator amplifying IL-1 production and sustaining the inflammatory cascade [3,58]. Understanding this cytokine crosstalk could provide insight into the continuum from systemic fetal inflammation to cerebral sequelae. Moreover, prospective studies are warranted to compare IL-6 levels in newborns who subsequently develop bacterial meningitis with those who do not, to evaluate its predictive potential for central nervous system involvement. Finally, integrating multiplex PCR techniques for simultaneous detection of bacterial pathogens in blood and cerebrospinal fluid, alongside cytokine profiling, would enhance diagnostic precision and allow a more refined characterization of the host–pathogen interaction in early neonatal infection.

Additionally, the integration of IL-6 monitoring into clinical protocols may facilitate more personalized care approaches, allowing healthcare providers to adapt interventions based on individual inflammatory profiles. This evolving perspective underscores the importance of continued research into IL-6’s roles, as understanding its long-term implications could lead to more effective therapeutic strategies aimed at improving overall neonatal outcomes for preterm infants.

## 7. Conclusions

UC IL-6 has shown consistent associations with early neonatal outcomes, including sepsis, respiratory distress and HIE, underscoring its potential as an early warning biomarker. Its rapid elevation in response to intrauterine inflammation makes it a valuable candidate for guiding timely interventions.

A major challenge in integrating UC IL-6 into routine clinical practice is the lack of standardized assays and reference intervals. Moreover, GA, labor status, and perinatal conditions significantly influence baseline IL-6 levels, underlining the need for stratified reference ranges. Establishing standardized methodologies and GA–specific cut-offs will be essential to enhance reproducibility, allow meaningful inter-study comparisons, and ultimately support the integration of IL-6 into risk stratification algorithms for neonatal outcomes.

In conclusion, UC IL-6 holds great promise as a biomarker for neonatal outcomes, but its clinical utility will only be fully realized once standardized assays and well-defined reference intervals become available.

## Figures and Tables

**Table 1:** Summary of selected studies presenting key evidence on UC IL-6 values in the diagnosis of EONS.

Study	Type of Study	Population	Key Findings	Cut-Off IL-6 (pg/mL)	Sensitivity (%)	Specificity (%)	Compared Markers	IL-6 vs. Others
**Basu et al. (2012)** [34]	Prospective cohort	93 newborns	IL-6 ≥40.5 pg/mL had 92.3% sensitivity, 90.5% specificity: better than traditional screening	40.5	92.3	90.5	Traditional screen	Outperformed standard sepsis screening
**Cernada et al. (2012)** [33]	Observational	177 preterm newborns	IL-6 AUC 0.88 vs. CRP 0.70 in EONS diagnosis				CRP	IL-6 superior to CRP
**Yuan et al. (2025)** [37]	Cohort	53 preterm newborns	IL-6 ≥250.5 pg/mL gave 90% sensitivity, 82% specificity	250.5	90	82	None	Effective standalone marker
**Chowdhary et al. (2020)** [42]	Observational	120 newborns	IL-6 more sensitive than CRP for EONS; better early marker	20	62.3		CRP	More sensitive than CRP (27.5%)
**Liu et al. (2020)** [29]	Diagnostic model	32 newborns	IL-6 had perfect AUC with CRP, PCT, serum amyloid A (AUC = 1.0)		100	100	CRP, PCT, serum amyloid A	Similar diagnostic value to other markers
**Steinberger et al. (2014)** [40]	Diagnostic evaluation	100 newborns	IL-6 showed good accuracy; PCT combo improved specificity	15.85	73.7	84.2	PCT	Combo with PCT raised specificity to 91.7%
**Yoon et al. (2000)** [39]	Case–control	94 newborns	IL-6 ≥17.5 pg/mL correlated with funisitis and sepsis	17.5	70	78	None	Effective standalone indicator
**Eichberger & Resch (2022)** [30]	Systematic Review	>3200 newborns (review)	IL-6 typical cut-off >30 pg/mL; best used with CRP	>30 (typical)	42.1–100	43–100	CRP (combo)	Combo raised sensitivity, reduced specificity
**Fadilah et al. (2022)** [38]	Prospective cohort	40 newborns	IL-6 ≥16.4 pg/mL → 5.5× higher risk of sepsis	16.4			None	No direct comparison
**Hatzidaki et al. (2005)** [36]	Prospective observational	89 newborns	Cut-off ≥28 pg/mL → 97% sensitivity, 91% specificity	28	97	91	CRP	Superior to CRP in early detection

**Table 2:** Diagnostic performance of IL-6 in EONS by sample type and timing.

Study	Sample Type	Timing of Sampling	Cut-Off (pg/mL)	Sensitivity (%)	Specificity (%)
**Basu et al., (2012)** [34]	UC blood	Immediately after delivery	40.5	92.3	90.5
**Cernada et al., (2012)** [33]	UC blood	At birth	255.87	90	87.4
**Yuan et al., (2025)** [37]	UC blood	At birth	250.5	90	82
**Fadilah et al., (2022)** [38]	UC blood	At birth	16.4	—	— (RR 5.54 for EONS)
**Steinberger et al., (2014)** [40]	Peripheral venous blood	Within first 24 h	15.85	73.7	84.2

**Table 3:** Overview of key studies exploring the relationship between UC IL-6 and respiratory complications.

Study Name	Type of Study	Population (n)	Key Findings	Cut-Off	Sensitivity	Specificity
**Rocha et al. (2012)** [43]	Prospective cohort	150 preterm newborns	Cord blood IL-6 associated with BPD in SGA pre-terms.	Not specified	Not specified	Not specified
**Ozalkaya et al. (2016)** [44]	Prospective observational	84 preterm newborns	IL-6 >26.7 pg/mL predicts RDS; >37.7 pg/mL predicts death in FIRS infants.	26.7 pg/mL (RDS), 37.7 pg/mL (death)	70% (RDS), 78.6% (death)	85% (RDS), 60% (death)
**Sorokin et al. (2014)** [46]	Secondary analysis of RCT	400 preterm newborns	IL-6 >75th percentile associated with increased RDS and CLD, but not significant after adjusting for GA.	>75th percentile	Not specified	Not specified
**Satar et al. (2008)** [45]	Case–control	83 preterm newborns	PPROM newborns had higher maternal serum IL-6; no significant difference in cord IL-6 levels.	Not specified	Not specified	Not specified
**Takahashi et al. (2010)** [3]	Prospective observational cohort study	224 newborns	Higher IL-6 levels were significantly associated with the presence of RDS in preterm infants.	Not clearly defined	Not specified	Not specified

**Table 4:** Descriptive summary of studies evaluating cord blood IL-6 as a biomarker in neurologic outcome.

Study	Population	IL-6 Sample Source	IL-6 Cut-Off or Range	Neurological Outcome(s)	Main Findings
**Goepfert et al. (2004)** [47]	400 preterm newborns	Umbilical cord blood	≥107.7 pg/mL	PVL	High IL-6 strongly predicted PVL (OR~30)
**Sorokin et al. (2014)** [46]	400 newborns (RCT follow-up)	Umbilical cord blood	>75th percentile	Bayley MDI or PDI < 70	No significant link with neurodevelopment after adjustment
**Musilova et al. (2018)** [24]	171 women with PPROM (34–37 weeks)	Umbilical cord blood	Defined FIRS: elevated IL-6 + histopathology	IVH (grades I–II)	FIRS linked to higher IVH and EONS
**Wolfsberger et al. (2020)** [49]	Preterm newborns with FIRS	Umbilical cord blood	FIRS defined as IL-6 > 11 pg/mL	Altered cerebral oxygenation; no acute injury	FIRS altered cerebral oxygen extraction, no brain injury increase
**Toorell et al., (2024)** [48]	150 term newborns	Umbilical cord blood	No defined cut-off value	Development of HIE	IL-6 levels were higher in HIE II/III versus HIE I

**Table 5:** Summary table of studies reporting UC IL-6 values and NEC.

Study	Study Design	Population & N	IL-6 in Cord Blood	Association with NEC	Cut-Off Value	Statistical Significance
**Goepfert et al. (2004)** [47]	Prospective cohort	Pregnant women with preterm labor (<35 weeks), N = 239	Elevated IL-6 (>11 pg/mL) significantly associated with adverse neonatal outcomes including NEC	Yes—elevated IL-6 linked with NEC (statistical significance reported)	11 pg/mL	Yes
**Satar et al. (2008)** [45]	Prospective observational	Newborns with PPROM (29–35 weeks GA), N = 83 (42 PPROM, 41 control)	No significant difference between PPROM and control; not associated with NEC	No—IL-8 was associated with NEC, not IL-6	Not applicable	No
**Sorokin et al. (2014)** [46]	Secondary analysis of RCT data	Newborns from pregnancies at risk for PTB, N = 400	IL-6 >75th percentile associated with NEC in <32 weeks GA subgroup, but not in full cohort	Partially—only in subgroup <32 weeks GA	75th percentile (not numerically defined)	Yes, in <32 weeks subgroup

**Table 6:** Summary table of studies reporting umbilical cord IL-6 values and neonatal mortality.

Study	Population & N	IL-6 Cut-Off	Mortality Rate	Statistical Findings
**Hofer et al. (2013)** [51]	Preterm infants, N = 176	>11 pg/mL	Significantly higher in FIRS group	*p* = 0.004; r = 0.411 (*p* < 0.001)
**Ozalkaya et al. (2016)** [44]	Preterm infants, N = 160	>11 pg/mL	19.3% (FIRS) vs. 1.9% (no FIRS)	*p* < 0.001
**Nomiyama et al. (2023)** [17]	Preterm infants 22–33 weeks, N = 330	FIRS + histology	Part of composite adverse outcome	aOR 6.84–7.17 (*p* < 0.001)
**Satar et al. (2008)** [45]	PPROM newborns, N = 83	Not defined	No significant difference	*p* > 0.05

**Table 7:** UC IL-6 cut-off values by gestational age group and assay method.

Study	GA Group	Delivery Context	Assay Platform	Cut-Off (pg/mL)	Cut-Off Origin
Basu et al., 2012 [34]	Mixed preterm	Mixed	ELISA	40.5	Derived
Cernada et al., 2012 [33]	<32 weeks	Mostly with labor/IAI	Chemiluminescent	255.8	Derived
Hatzidaki et al., 2005 [36]	<34 weeks, PPROM	Inflammation-associated	ELISA	28	Derived
Yuan et al., 2025 [37]	28–34 weeks	Mixed	ELISA	250.5	Validated
Fadilah et al., 2022 [38]	Late preterm	Mixed	ELISA	16.4	Derived
Sorokin et al., 2014 [46]	Mixed (preterm + term)	Mixed	ELISA	75th percentile	Derived

## Data Availability

Not applicable.

## Abbreviations

The following abbreviations are used in this manuscript:

AUC	Area under the receiver operating characteristic curve
BPD	Bronchopulmonary dysplasia
CLD	Chronic lung disease
CRP	C-reactive protein
ELISA	Enzyme-linked immunosorbent assay
EONS	Early-onset neonatal sepsis
FIRS	Fetal inflammatory response syndrome
GA	Gestational age
HIE	Hypoxic–ischemic encephalopathy
IAI	Intra-amniotic inflammation
IL-6	Interleukin-6
IVH	Intraventricular hemorrhage
MDI	Mental Development Index
MIAC	Microbial invasion of the amniotic cavity
NEC	Necrotizing enterocolitis
PCT	Procalcitonin
PPROM	Preterm premature rupture of membranes
PDI	Psychomotor Developmental Index
PVL	Periventricular leukomalacia
RCT	Randomized control trial
RDS	Respiratory distress syndrome
RR	Risk ratio
UC	Umbilical cord
